# First person – Jenna Villman

**DOI:** 10.1242/dmm.053219

**Published:** 2026-10-16

## Abstract

First Person is a series of interviews with the first authors of a selection of papers published in Disease Models & Mechanisms, helping researchers promote themselves alongside their papers. Jenna Villman is first author on ‘ Pharmacokinetics and efficacy of tank-water-administered dabrafenib, a BRAF inhibitor, in a zebrafish model of melanoma’, published in DMM. Jenna is a Project Researcher in the lab of Ilkka Paatero at Turku Bioscience Centre, Turku, Finland, investigating zebrafish model development for studying disease mechanisms and therapies.

**Figure:**
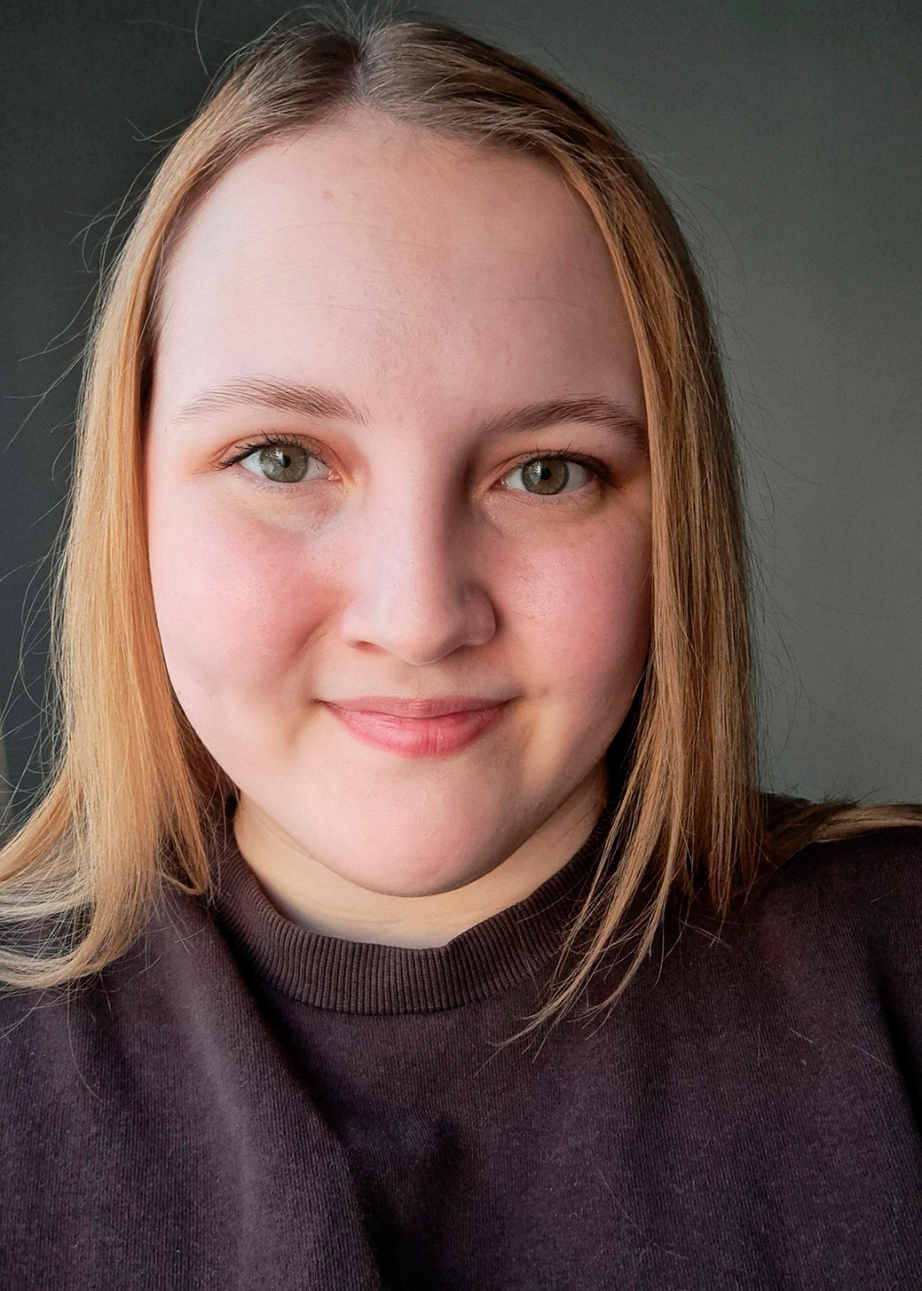
Jenna Villman

**Who or what inspired you to become a scientist?**

I have always been interested in the things that we do not yet know. Even as a small child, I wondered why we could not figure some things out. I became particularly interested in biomedical science because I had several personal experiences with disease. I wanted to do something that could have a real impact. Now, as a scientist, I do not only want to understand how but also why things work the way they do.

**What is the main question or challenge in disease biology you are addressing in this paper? How did you go about investigating your question or challenge?**

Zebrafish are commonly used as disease models in biomedical research. However, knowledge on the pharmacokinetics of small-molecule inhibitors in zebrafish is limited, and long-term treatment of adult zebrafish with the inhibitors can be challenging. In this study, we addressed these research gaps by administering a BRAF inhibitor, dabrafenib, directly through tank water to adult zebrafish in a melanoma model. We investigated and measured its absorption, elimination, metabolism, mechanism of action and efficacy. We also performed confirmatory studies using zebrafish cell culture.

When we treated zebrafish with melanoma, dabrafenib reduced the size of the tumours – in some cases, to the point where the tumours had almost completely disappeared

**How would you explain the main findings of your paper to non-scientific family and friends?**

Dabrafenib is a cancer drug used to treat a type of skin cancer called melanoma. We wanted to find out whether the drug would work in zebrafish in a similar way to how it works in humans when it is administered through tank water. We found that the fish took up the drug, processed it and distributed it throughout their bodies in a similar way to that observed in humans. When we treated zebrafish with melanoma, dabrafenib reduced the size of the tumours – in some cases, to the point where the tumours had almost completely disappeared. We also showed that the drug works in zebrafish through the same biological process that makes it effective in humans. These findings were confirmed in experiments with zebrafish melanoma cells. Overall, our study shows that giving dabrafenib through tank water is a practical way to treat adult zebrafish and provides useful information for determining how much of the drug is needed for treatment.

**What are the potential implications of these results for disease biology and the possible impact on patients?**

In adult zebrafish studies, drugs are often administered directly into the abdominal cavity or mixed with the feed. We found that long-term dosing through tank water is a feasible and practical way to administer drugs, which can facilitate future studies using adult zebrafish as disease models. Our study also provided information about pharmacokinetic aspects in zebrafish, which can help researchers select more rational dosing strategies in future experiments. In the longer term, better understanding of drug dosing and exposure in disease models can contribute to more reliable preclinical research and, potentially, to the development of improved treatments for patients.

**Figure:**
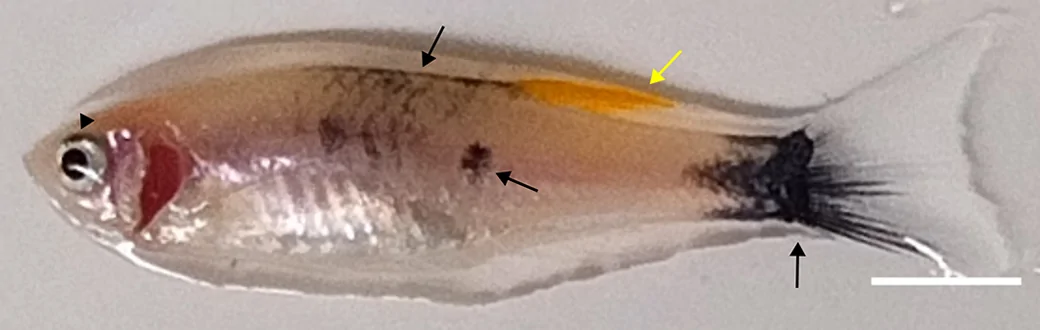
**Zebrafish melanoma model showing melanoma in the skin (black arrows) and eye (black arrowhead) alongside xanthophore tumours (yellow arrow), which have no known human counterpart.** Scale bar: 5 mm.

**Why did you choose DMM for your paper?**

The scope of DMM, with a focus on disease mechanisms and therapies through disease modelling, closely aligns with our paper. We appreciate the journal's focus on translational research and its interdisciplinary approach to different diseases and disease models, including zebrafish. In addition, we wanted to publish our work in a well-respected, peer-reviewed and open-access journal.

**Given your current role, what challenges do you face and what changes could improve the professional lives of other scientists in this role?**

As a project researcher, one of the biggest challenges is uncertainty about my future role. Positions are often project-based and relatively short, sometimes less than 6 months, which can make it difficult to plan for the future. In addition, the funding situation of the research group affects the availability of positions. One change that could improve the professional lives of other scientists in similar roles would be to offer longer contracts, which would provide greater stability and allow researchers to plan their careers and research more effectively.

**What's next for you?**

During the study, we observed pigmented tumours in the eye that responded to BRAF inhibitor treatment. These tumours may represent a rare melanoma subtype called conjunctival melanoma. As there are currently no established genetic *in vivo* models or approved targeted therapies for this disease, further characterization and development of this model are needed. I have applied for a PhD position to continue working on the findings.

**Tell us something interesting about yourself that wouldn't be on your CV**

I am the oldest of four sisters, and I think that has shaped who I am today as a person. Being a big sister has taught me a lot about responsibility, patience and adaptability, as well as an appreciation for different personalities and perspectives.
